# Case reports: Using Good Psychiatric Management (GPM) conceptualizations in the dimensional assessment and treatment of personality disorders

**DOI:** 10.3389/fpsyt.2023.1186524

**Published:** 2023-07-26

**Authors:** Martin Blay, Ines Benmakhlouf, Miguel Duarte, Nader Perroud, Christian Greiner, Patrick Charbon, Lois Choi-Kain, Mario Speranza

**Affiliations:** ^1^ADDIPSY, Outpatient Addictology and Psychiatry Center, Santé Basque Développement Group, Lyon, France; ^2^Lyon Est Faculty of Medicine, Claude Bernard University Lyon 1, Lyon, France; ^3^Department of Mental Health and Psychiatry, Service of Psychiatric Specialties, University Hospitals of Geneva, Geneva, Switzerland; ^4^Department of Psychiatry, University of Geneva, Geneva, Switzerland; ^5^Consultative Psychiatry and Crisis Intervention Department, University Hospitals of Geneva, Geneva, Switzerland; ^6^McLean Hospital, Gunderson Personality Disorders Institute, Belmont, MA, United States; ^7^Department of Psychiatry, Harvard Medical School, Boston, MA, United States; ^8^Versailles Hospital Center, University Department of Child and Adolescent Psychiatry, Le Chesnay, France; ^9^Centre de recherche en Epidémiologie et Santé des Populations Team “DevPsy”, Université de Versailles Saint-Quentin-en-Yvelines (UVSQ), INSERM, Université Paris-Saclay, Villejuif, France

**Keywords:** case report, Good Psychiatric management, GPM, borderline personality disorder, narcissistic personality disorder, obsessive-compulsive personality disorder, personality assessment

## Abstract

Good Psychiatric Management (GPM) is a generalist clinical management approach for borderline personality disorder that incorporates common ingredients of good standard care for any psychiatric diagnosis with what works from prevailing specialist psychotherapies. Similar to all validated therapies for BPD, it relies on a specified formulation of the disorder' symptoms as arising from *interpersonal hypersensitivity*, to dynamically describe typical patterns of daily self- and interpersonal issues that drive the instability that defines the general personality dysfunction characteristic of the disorder. Recent adaptations of GPM have been proposed for narcissistic personality disorder and obsessive-compulsive personality disorder, with development of similar dynamic models for both (*intrapsychic coherence model* and *model of overcontrol*). New dimensional models of personality disorder diagnosis have been developed to address limitations of categorical approach, but the incorporation of these models into usage in the delivery of clinical services (where categorical approach remains the most used) is limited. This paper describes an adaptation of GPM to two cases of personality disorder that illustrate the usefulness of GPM models for dynamic representation of complex daily fluctuations in internal psychic coherence and interpersonal functioning. Specialist psychotherapies will never meet the demands of public health needs to treat personality dysfunction, and incorporation of new dimensional models of diagnosis are needed for treatments that can provide a minimal standard of care for providers and patients.

## 1. Introduction

Good Psychiatric Management (GPM), is a structured clinical approach to treatment of borderline personality disorder (BPD) that provides a less specialized pragmatic means of providing treatment to those with BPD, a condition that affects around 1.6% of the general population and consists in significant difficulties in emotion regulation, identity and interpersonal relationships, with impulsive, suicidal and para-suicidal behaviors; symptoms which are conceptually driven by a fear of abandonment and rejection (1). It is based on a dynamic model called the interpersonal hypersensitivity (also named the interpersonal coherence model), which provides a framework for understanding the fluctuating self- and interpersonal issues patterns prototypical of BPD (2). Following this formulation informed by psychodynamic, cognitive behavioral, and medical models of treatment, individuals with BPD, when confronted to the perception of being abandoned or rejected, experience acute distress, interpersonal conflicts, anger outbursts, non-suicidal self-injury behaviors (NSSIs), or sometimes suicide attempts. Given its efficacy for treating BPD symptoms (3, 4), recent GPM adaptations have been proposed for two other personality disorders, narcissistic personality disorder (NPD) (5) and obsessive-compulsive personality disorder (OCPD) (6), for which no evidence based treatments have been adequately developed and tested empirically.

People affected with pathological narcissism or NPD are fundamentally characterized by difficulties regulating self-esteem (7). Indeed, according to the GPM intrapsychic coherence model (5), patients with NPD have a fragile and idealized sense of self, so that when these patients are confronted to real or perceived self-esteem threats, they react with desperate efforts to regain self-esteem (including aggressive devaluation of others as well as efforts to re-inflate one's self). Like with BPD, those with NPD also experience oscillations between threatened states and states of aloneness. On the other hand, OCPD—the most common personality disorder in the western world (8)—has been described by several experts as a condition defined by an intolerance to loss of control and rigid perfectionism (6). According to the GPM model of overcontrol, when confronted to internal or external threats to order or control, patients may start being anxious or angry, and resorting to intense efforts to overcontrol the situation, which can also lead to emotion dysregulation (9), interpersonal issues (10), self-harm and suicidal behaviors (11). The corresponding triggers for these three disorders are listed in Table 1.

**Table 1:** Relevant triggers in borderline, narcissistic and obsessive-compulsive personality disorders according to GPM.

**Disorder**	**Model**	**Trigger**
Borderline personality disorder	*Interpersonal coherence model*	Real or imagined rejection and abandonment (2)
Narcissistic personality disorder/Pathological narcissism	*Intrapsychic coherence model*	Real or imagined self-esteem threats (5)
Obsessive-compulsive personality disorder	*Model of overcontrol*	Internal or external threat to perfection, order, or Control (6)

Clinicians treating these three disorders are confronted with symptomatic overlap between them, in terms of emotional, behavioral and interpersonal dysregulation. Given that these aspects are only emphasized in the BPD criteria list, and are thus thought to be classic borderline symptoms, there is a risk that clinicians will focus excessively on these externalized symptoms (and thus on BPD diagnosis), which may lead them to underestimate underlying narcissistic or obsessive-compulsive personality problems that represent different core psychopathological issues. This can be linked to the recent paradigm shift in the personality disorder field. Indeed, in the last decade, categorical approach of personality disorder has been criticized, notably due to clinical heterogeneity within categories (12). Dimensional models (e.g., Alternative Model of Personality Disorders of the DSM-5 or the personality disorders model of the International Classification of Disease 11th version) have been developed, aiming at better describing personality pathology using the association of a general factor (described as the level of personality functioning) with several personality traits (negative affectivity, antagonism/dissociality, detachment, disinhibition, anankastia, psychoticism). In these models, personality functioning is assessed on the level of self and interpersonal functioning and is thought to be the core changing aspect over time (13). On the other hand, personality traits are used to describe specific characteristics of one's personality and are thought to be more stable. Moreover, several works have suggested that BPD criteria may be the strongest markers of general personality functioning (13, 14), which may explain the common clinical aspects found for the three disorders mentioned above, as they may be related more to the severity of the personality dysfunction than to an underlying psychopathology of BPD.

While these new models offer novel possibilities in terms of diagnostic assessment, pragmatic means of incorporating them into the delivery of effective clinical services are crucial in order to make such changes beneficial for most patients and most clinicians regardless of theoretical orientation, discipline, or clinical context. Indeed, these models have troubles implementing in clinical services (15) and generalist clinicians, who are not specialized in personality disorders, continue to use the categorical approach, the lack of familiarity with dimensional models mainly concerning the personality traits in criteria B. Indeed, these traits are only descriptive, and represent rigids modalities of functioning, in the sense that they hardly change over time (13). Moreover, these traits are also non-specifics, given that the expression of these traits can be really different in one patient or another. Overall, due to both this rigidity and non-specificity, it seems coherent to assume that these traits are not representative of the complex daily fluctuations in internal psychic coherence and interpersonal functioning dynamics characteristics of PDs. If we want to make the dimensional models practicable, we need to provide a framework for dynamically incorporating personality traits into a paradigm that integrates these complex fluctuations. Otherwise, discussion and disclosure of these traits in the diagnostic process may be experienced as distant and abstract by patients who need a more holistic and experience-centered narrative representation of their suffering to which they can relate in their daily life. In this context, there is a need to develop a synthesis between existing categorical treatments and dimensional approaches to understanding personality dysfunction.

**Figure 1:**
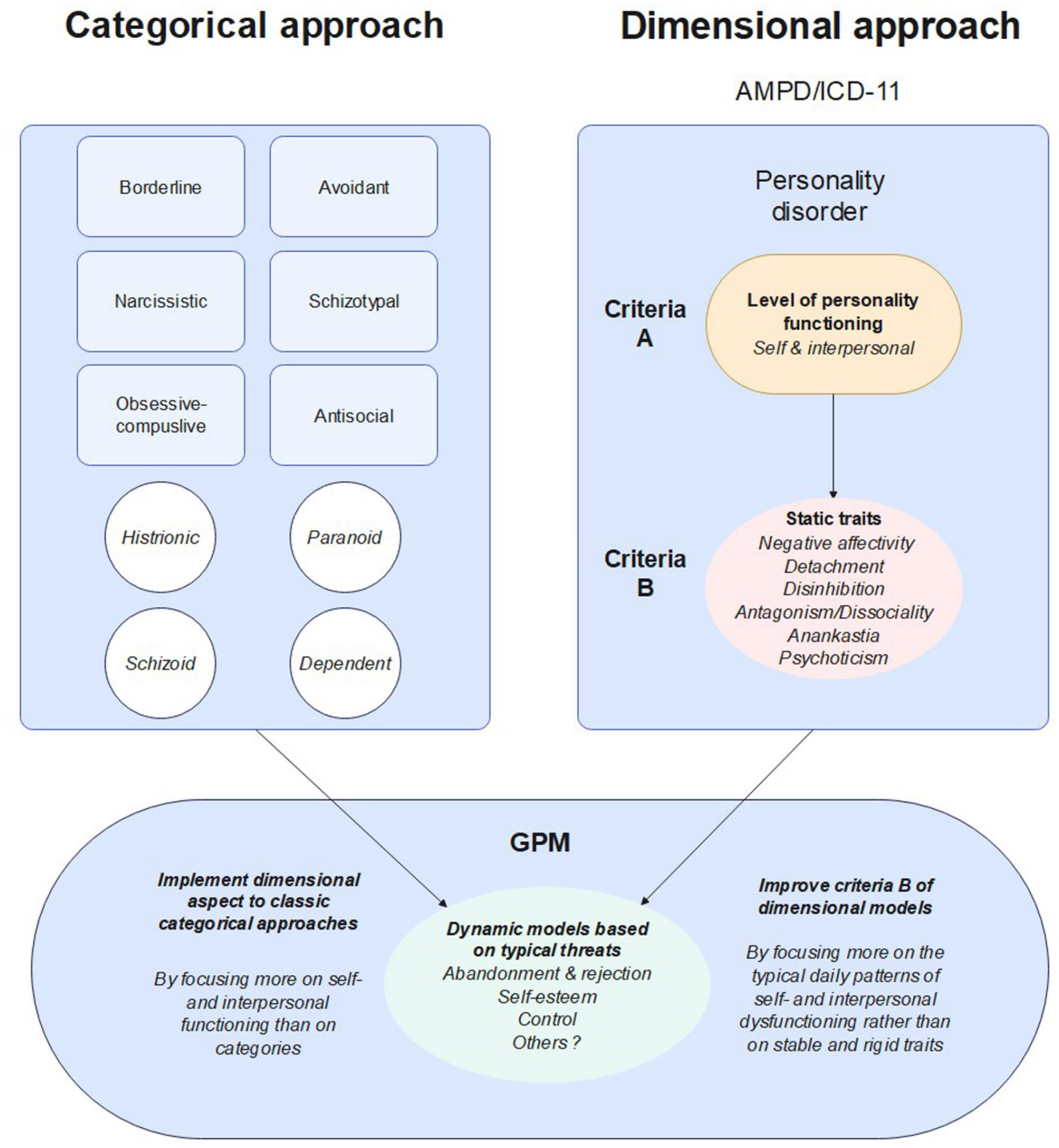
Representation of how GPM models may be implemented in categorical and dimensional approaches.

GPM, like the other major specialist treatment models, is based on a central formulation of how personality disorders work. Its broadening formulations of personality as stress sensitive disorders incorporate different prototypes of fight of flight responses embedded in the interpersonal and self-management model. Since it incorporates common factors of therapies designed for PD, with a medicalized framework of care that meets standards of national guidelines, GPM's adaptation of dimensional approaches to PD may be useful to make them more relevant to most clinicians and clinical context. GPM's formulations offer easy-to explain and personally resonant stories of core clinical problems related to PDs that enable clinicians and patients to best understand problem areas and create foci of change and intervention. By focusing more on specific threats to personality functioning and less on discrete diagnostic categories, we believe that such approach may help generalist clinicians, who are new to using dimensional models of PD, to incorporate a more dimensional assessment of personality in their daily practice, with a focus on self and interpersonal functioning. Moreover, these models may also be useful to help specialists using dimensional models to incorporate representations that meaningfully lend greater coherence for patients and clinicians to better understand and discuss their daily experience. Here, we propose a GPM based model to incorporate dimensional modes of thinking about PDs and to provide a dynamic but descriptively clear way of understanding self- and interpersonal dysfunction in an individual with personality disorder. A global representation of this proposition can be found in Figure 1.

To present how this model may be useful in clinical practice, we describe two clinical cases, initially addressed for BPD treatment, whose diagnosis and treatment have been reoriented using a GPM-based clinical investigation and psychometric dimensional measures.

## 2. Case description 1: narcissistic self-esteem fragility as an expression of severe personality disorder with core detachment and negative affectivity in a patient diagnosed with BPD and three other personality disorders

Mr. A is a 25-years old patient addressed by his psychiatrist for the initiation of a specialized psychotherapy for a diagnosis of borderline personality disorder. Regarding family psychiatric history, he reports a history of depression in his mother with several suicide attempts. Apart from a diagnosis of attention deficit hyperactivity disorder and several learning disabilities in his childhood, he had no medical or psychiatric history or a long-term follow-up. During his 15–20 first years of life, he described himself as “very confident” with a very high self-esteem (“before 19 I felt like God, I could manage to do everything I wanted and should do”). In 2015–2016, he reports the appearance of psychosomatic symptoms secondary to a romantic breakup, resulting in a partial withdrawal from social relationships. Difficulties increased in 2017–2018 with the onset of panic attacks and the progressive aggravation of his social withdrawal, with a second aggravation in 2020 where he was hospitalized for “dissociation and psychotic symptoms.” At that time, he presented a severe anxiety syndrome and severe difficulties in emotion regulation, resulting in addictive behavior and self-cutting. In his first hospitalization, he received a diagnosis of BPD, generalized anxiety disorder and post-traumatic stress disorder. He had another hospitalization in the same year for the same motives and was then admitted in our unit for the treatment of his BPD and his substance abuse disorder.

At his arrival, he was evaluated with a semi-structured interview (Structured Clinical Interview for DSM-5 Personality Disorders (SCID)) for borderline, narcissistic, avoidant, obsessive-compulsive, schizotypal and antisocial personality disorders, and he also completed the following self-report scales: Borderline Symptom List −23 items (BSL-23), Zanarini Scale for Borderline Personality Disorder (ZAN BPD) and Pathological Narcissism Inventory-Brief form (PNI-B). His results can be found in Table 2. Overall, he met cutoffs for borderline, narcissistic, obsessive-compulsive, and antisocial personality disorders. Antisocial behaviors found at the SCID interview mainly consisted in impulsive, aggressive behaviors, and reckless disregard for his own safety between 2017 and 2020. He had very high symptom ratings of BPD according to BSL-23 (16), severe BPD symptoms according to the ZAN-BPD scale (17), with a mean level of narcissistic grandiosity globally similar but a mean level of narcissistic vulnerability of 0.5 to 1 point higher than those obtained in male patients from the general population in the validation study of the PNI-B (18). On a dimensional level, using the ICD-11 criteria, he was clinically assessed as having a severe personality disorder, with significant levels of negative affectivity, detachment, dissociality, and anankastia.

**Table 2:** Main psychometric results of Mr. A.

**Scales**	**Subscales**	**Scores (range)**
**SCID**		
	Borderline	9 (0–9)
	Narcissistic	5 (0–9)
	Avoidant	3 (0–7)
	Obsessive-compulsive	5 (0–8)
	Schizotypal	1 (0–9)
	Antisocial	5 (0–7)
BSL-23		3.04 (0–4)
ZAN-BPD		25 (0–36)
**PNI-B**		
	Grandiosity	3 (0–5)
	Vulnerability	2.9 (0–5)

BSL-23, Borderline Symptom List − 23 items; PNI-B, Pathological Narcissism Inventory—Brief form; SCID, Structural Clinical Interview for DSM-5 Personality Disorders; ZAN-BPD, Zanarini Scale for Borderline Personality Disorder.

Given the diagnosis of BPD, he participated in our GPM-based psychoeducation group. In one group session, the topic of abandonment emerged. He told us that he felt that BPD was insufficient for the description of his experience (“I recognize myself in the emotional troubles and self-harm behaviors, but abandonment is not a big issue for me”). This discussion started a shared reflection on the main threats he identified as relevant, particularly self-esteem. He shared:

“Growing up with my mother was really tough, we were often on our own, so I had to build on a self-image of being invincible because I had to manage everything. This made it possible to escape the fact that I didn't know anything about who I really was deep inside. For years, it worked. I was a great athlete, I had a large group of friends, everything was going well, until this break-up. But when my girlfriend broke with me, it was like… I felt like crap. She had been the only one supporting me. And then, I couldn't deal with the fact that I had been rejected. I went out less and less with my friends. I couldn't tolerate that they saw me differently than they had always seen me, great at everything. Progressively, I saw myself managing my life horribly, plagued with self-critical thoughts, comparing myself to what I had been, what I should be and what I was capable of being. And 1 day, I was unable to manage a water leak. It's silly, but it lasted for weeks, and from there I started to feel such a deep, violent sense of shame about the failure that my life was becoming compared to what I was and what I should have become. I became very anxious, to the point of cutting myself to survive this feeling of collapse. Today, I feel a bit better, but I don't know who I am, I'm lost. Very often, this feeling of shame kicks in again, and that's always tough to manage.”

These interviews underlined the narcissistic vulnerabilities the patient reported in both the SCID interview and the PNI. We therefore chose to adopt a dimensional approach focusing on hypersensitivity to threats to self-esteem. We introduced the intrapsychic coherence model to the patient and, after several psychoeducation sessions, he told us that this dysregulation of self-esteem approach really described what he was feeling. This model also helped us understand the obsessive-compulsive behaviors found in the SCID interview, that were described by the patient as ways to keep control over others' perception and judgment, through high perfectionism, rigidity, and hyper-conscientiousness. Moreover, this made it possible to start case management work focusing on everyday events with the utilization of the different states narcissistic patients can face. Second, we started to establish goals for therapy, mainly self-esteem stabilization through confrontation with reality and re-evaluation of life goals rooted in reality rather than in his grandiose ideals for himself, with the aim of developing a more stable and coherent sense of identity and self-direction. We reoriented the care on GPM treatment focusing on narcissistic self-esteem dysregulation. We also started a work focused on social life to increase interpersonal functioning, with efforts to expose him to the possibility to be seen as fallible and vulnerable by his significant others. Notably, the corrective experience of seeing he could survive these moments of being seen as less than incredible and more human allowed him to diminish his avoidance.

## 3. Case description 2: fear of losing control as an expression of severe personality disorder with core anankastia in a patient diagnosed with BPD and OCPD

Mrs. B is a 25-year-old woman also referred to a specialized psychotherapy for a diagnosis of borderline personality disorder. Regarding psychiatric family history, she reports a depressive history in both side of the family. She has no reported history of trauma. She went to college, but her medical issues made her dropout. She is quite isolated, reporting few significant relationships. Her psychiatric history started in 2020 with the emergence of self-harm behaviors, which led to a short stay in hospital at the end of 2020 and again in 2021. A diagnosis of BPD has been made in 2021, given the self-harm behaviors and significant emotional dysregulation. Concerning the diagnosis, she explained that while she can relate to the emotion dysregulation and related aspects, abandonment is not a central issue.

At her arrival in our unit, she also completed the semi-structured interview and the self-report questionnaires. Her results can be found in Table 3. Overall, she was positive for borderline and obsessive-compulsive personality disorder, she had moderate BPD symptoms according to BSL-23, mild BPD symptoms according to ZAN-BPD scale, and she had a lower mean level of narcissistic grandiosity and a 0.5 to 1 point higher mean level of narcissistic vulnerability to those obtained in female patients from the general population in the validation study of the PNI-B. Considering her self-critical nature, the team also administered the Pathological Obsessive-compulsive Personality Scale (POPS). She had a higher mean total score (and higher mean emotional overcontrol, maladaptive perfectionism, reluctance to delegate and difficulty with change sub scores) than undergraduate students from the study validation sample (19). Finally, using ICD-11 criteria, she was also clinically assessed as severe personality disorder, with a core trait of anankastia and concomitant significant levels of negative affectivity and detachment.

**Table 3:** Main psychometric results of Mrs. B.

**Scales**	**Subscales**	**Score (range)**
**SCID**		
	Borderline	7 (0–9)
	Narcissistic	0 (0–9)
	Avoidant	0 (0–7)
	Obsessive-compulsive	4 (0–8)
	Schizotypal	0 (0–9)
	Antisocial	0 (0–7)
BSL-23		1.52 (0–4)
ZAN-BPD		9 (0–36)
**PNI-B**		
	Grandiosity	2.25 (0–5)
	Vulnerability	3.00 (0–5)
**POPS**		
	Total	200 (49–294)
	Rigidity	45 (15–90)
	Emotional over-control	30 (7–42)
	Maladaptive perfectionnism	55 (12–72)
	Reluctance to delegate	37 (8–48)
	Difficulty with change	37 (8–48)

BSL-23, Borderline Symptom List-23 items; PNI-B, Pathological Narcissism Inventory—Brief form; POPS, Pathological Obsessive-compulsive Personality Scale; SCID, Structural Clinical Interview for DSM-5 Personality Disorders; ZAN-BPD, Zanarini Scale for Borderline Personality Disorder.

At clinical assessment, she first presented distant, with low emotional expression. She described “blockages” due to fear of the unknown and of losing control. She reported high personal moral standards, with feelings of guilt about not having a personal and professional life (“I'm just a lazy person who procrastinates, I'm irresponsible, I should be working instead of enjoying myself”). She described herself as stubborn, with a severe performance anxiety. While she reported feeling mostly emotionally detached, she sometimes experienced emotional storms where she was overwhelmed by anger, guilt or fear. During these emotional storms, she reports use of self-harm and substance abuse to help her regulate. Interpersonal difficulties and social withdrawal were reported to be caused by mistrust, high expectations for her and for others to meet her moral standards, and fear of losing control over the relation.

Given that clinical presentation and psychometric results, we chose to reconsider the obsessive-compulsive traits underlined by the SCID interview and the POPS, and we once again chose to adopt a dimensional approach, this time focusing on the need for control. Thus, we started to use the model of overcontrol to present her our hypothesis. She really appreciated this “diagnostically decentered” approach, which made her able to start to open-up a little more in the next sessions, describing the state of paralysis she felt being into, with concomitantly the pain of being in her situation but also the tremendous fear of losing control if she tried to change it. Thus, we were able to read this state of paralysis and recent crises using the different states that obsessive-compulsive patients often go through. This GPM formulation resonated for her. She stated, “Although, as you have understood, I really do not wish to be put in a diagnosis, I must admit that this model really speaks to me.” We were able to start to better understand her usual difficulties in terms of her hypersensitivity to losing control. This helped her open-up more and more in the sessions. Finally, the dimensional assessment made it possible to reorientate the care to a GPM treatment focusing on fear of losing control, with an emphasis on building a sense of self not only relying on effort and moral values, but also on social inclusion and return to employment.

## 4. Discussion

We believe that these two cases underlie the potential interest of GPM models as a tool for dimensional assessment of PDs.

The recent arrival of new dimensional models led to a large paradigm shift in the personality disorder field. However, these models are struggling to spread to clinical practice, and many clinicians continue to use and to think in terms of categorical approach. In this context, we believe that GPM models may help overcome this issue by moving the focus on triggers and threats to dependency, self-esteem, and control in prototypical dynamics related to borderline, narcissistic, and obsessive-compulsive personality disorders. They allow generalist clinicians to have simple to use dimensional tools that may help them distinguish, in the different categorical PD diagnoses, the most relevant issues for the patient. In that sense, we believe that these models may be seen as simple and easy-to-be-trained-to dimensional models for generalist clinicians that could guide them in their diagnostic assessment.

GPM models may also be helpful to improve conventional dimensional models by implementing more dynamic and daily-life related representations of personality characteristics than the criteria B trait focused system. Despite their clinical utility, personality traits can be sometimes hard to understand and/or to explain to patients. Traits may also be thought as too static and distant from patients' everyday experiences. GPM models may mitigate this issue by providing a dynamic way of understanding patient's experience that's closer to what he/her feels, by focusing on simple affects and patterns of fight of flight to specific vulnerabilities, rather than on criteria or traits. This may be of great interest when considering the importance of the patient's feeling of being understood by the therapist in order to establish a productive therapeutic relationship (20).

Finally, using GPM as a frame for diagnostic assessment may also be useful in terms of treatment orientation. To date, no evidence-based treatments have been developed based on dimensional models. On the other hand, GPM has been proven to be useful to improve BPD symptoms (3, 4), and one could infer that it could also be effective in improving general personality dysfunction, given that BPD is thought to be a relevant marker of the latter (13), and given the improvement in interpersonal functioning in GPM trials and the overall focus on getting a life outside of treatment in GPM framework. In that sense, we believe that implementing GPM while improving it by incorporating a more dimensional frame would be useful to concomitantly implement evidence-based conceptualization and evidence-based treatment for PD.

## 5. Conclusion

These two cases illustrate the potential interest of GPM conceptualizations of PDs in terms of diagnostic assessment and psychoeducation. We believe that these models may be suitable for generalist clinicians to help them implement dimensional personality assessment in their daily practice and may also be helpful to improve the recent dimensional models, notably in terms of characteristics of personality dysfunction.

## Data availability statement

The original contributions presented in the study are included in the article, further inquiries can be directed to the corresponding author.

## Ethics statement

Ethical review and approval was not required for the study on human participants in accordance with the local legislation and institutional requirements. The patients/participants provided their written informed consent to participate in this study. Written informed consent was obtained from the participant/patient(s) for the publication of this case report.

## Author contributions

MB conducted initial assessment and clinical follow-up of the patients, conducted the literature review, wrote the first draft of the manuscript, and participated in the review of every version. IB conducted the administration and collection of the psychometric tools. MD, NP, CG, PC, and MS helped on the conceptualization of the report, helped conducting the literature review, revised the manuscript, and contributed to its completion. LC-K revised the manuscript and contributed to its completion. All authors read and approved the final manuscript.
